# An audit of treatment focussed BRCA1/2 mutation testing at an integrated Familial Cancer Clinic

**DOI:** 10.1186/1897-4287-10-S2-A49

**Published:** 2012-04-12

**Authors:** A Lewis, L Cicciarelli, D Pandey, CM Lovett, R Driessen, S Sawyer, MA Young, G Mitchell

**Affiliations:** 1 Peter MacCallum Familial Cancer Centre, Melbourne, Victoria, Australia

## Background

The growth of a personalised approach to cancer treatment including surgical risk management strategies and Parp Inhibitor trials for BRCA1/2 mutation carriers has lead to the Peter MacCallum Familial Cancer Centre experiencing a rapidly increased demand for expedited risk assessment appointments +/- BRCA1/2 mutation testing.

Expedited BRCA mutation screens are offered to individuals assessed eligible for publically funded testing and require these results in a defined time period to assist in their current cancer management.

The demand for expedited services places a burden on both the laboratory and clinic as patients need to be seen at short notice and have their genetic test take priority over routine tests.

## Aim

To better understand the clinical utility and mutation detection rate of expedited BRCA1/2 tests, in order to improve services and resources for this group of patients.

## Methods

A retrospective review of the Peter MacCallum Familial Cancer Centre’s BRCA1/2 mutation testing data from January 2007-April 2011 was performed. During this time period 119 patients were offered a treatment-focussed genetic test. The characteristics of the tested cohort were reviewed and analysed.

## Results

Numbers of referrals for expedited risk assessment and requests for treatment focussed BRCA1/2 mutation testing remained consistent between 2007 and 2009 although have significantly increased in 2010 with nearly a twofold increase.

Most patients referred had been recently diagnosed with breast cancer and were considering the option of breast conservation versus mastectomy. To assist them with this decision they were seeking advice about their genetic risk of developing a new primary breast cancer. In addition, referrals were also received to determine the eligibility of some patients for Parp Inhibitor trials.

A pathogenic mutation was detected in 20/119 (16.8per cent) of patients who had an expedited BRCA1/2 test. Further analysis about the pre and post testing treatment decisions is being analysed and will be presented.

## Conclusion

In 2010 a significant increase in demand for treatment focussed risk assessments and expedited BRCA1/2 tests was experienced by the Peter MacCallum Familial Cancer Centre. Further data will be presented on the characteristics of these cohorts and the clinical utility of expedited assessments.

